# A method to define the relevant ego-centred spatial scale for the assessment of neighbourhood effects: the example of cardiovascular risk factors

**DOI:** 10.1186/s12889-021-11356-w

**Published:** 2021-07-07

**Authors:** Jürgen Breckenkamp, Oliver Razum, Jacob Spallek, Klaus Berger, Basile Chaix, Odile Sauzet

**Affiliations:** 1 Department of Epidemiology and International Public Health, School of Public Health, Bielefeld University, Bielefeld, Germany; 2 Department of Public Health, Brandenburg University of Technology Cottbus-Senftenberg, Senftenberg, Germany; 3 Institute of Epidemiology and Social Medicine, University of Münster, Münster, Germany; 4 Sorbonne Université, Inserm, Institut Pierre Louis d’Epidémiologie et de Santé Publique, Nemesis research team, Paris, France

**Keywords:** Cardiovascular risk factors, Geographic distribution, Spatial scale

## Abstract

**Introduction:**

The neighbourhood in which one lives affects health through complex pathways not yet fully understood. A way to move forward in assessing these pathways direction is to explore the spatial structure of health phenomena to generate hypotheses and examine whether the neighbourhood characteristics are able to explain this spatial structure. We compare the spatial structure of two cardiovascular disease risk factors in three European urban areas, thus assessing if a non-measured neighbourhood effect or spatial processes is present by either modelling the correlation structure at individual level or by estimating the intra-class correlation within administrative units.

**Methods:**

Data from three independent studies (RECORD, DHS and BaBi), covering each a European urban area, are used. The characteristics of the spatial correlation structure of cardiovascular risk factors (BMI and systolic blood pressure) adjusted for age, sex, educational attainment and income are estimated by fitting an exponential model to the semi-variogram based on the geo-coordinates of places of residence. For comparison purposes, a random effect model is also fitted to estimate the intra-class correlation within administrative units. We then discuss the benefits of modelling the correlation structure to evaluate the presence of unmeasured spatial effects on health.

**Results:**

BMI and blood pressure are consistently found to be spatially structured across the studies, the spatial correlation structures being stronger for BMI. Eight to 22% of the variability in BMI were spatially structured with radii ranging from 100 to 240 m (range). Only a small part of the correlation of residuals was explained by adjusting for the correlation within administrative units (from 0 to 4 percentage points).

**Discussion:**

The individual spatial correlation approach provides much stronger evidence of spatial effects than the multilevel approach even for small administrative units. Spatial correlation structure offers new possibilities to assess the relevant spatial scale for health. Stronger correlation structure seen for BMI may be due to neighbourhood socioeconomic conditions and processes like social norms at work in the immediate neighbourhood.

**Supplementary Information:**

The online version contains supplementary material available at 10.1186/s12889-021-11356-w.

## Introduction

Structural deprivation models are frequently used to describe and analyse regional differences in health status and disease prevalences within and across populations. It is well-known that an individual’s neighbourhood affects health through complex pathways which, however, are not fully understood. A major challenge to be met by studies of neighbourhood effects on specific health outcomes is to describe and explain the geographic distribution of health outcomes and their spatial variability. It is relevant to investigate both the magnitude of spatial variations of health phenomena but also their spatial scale, i.e., whether they vary in space on a local or broader basis. Identifying the spatial scale over which health outcomes vary in space is important as it may have implications on the geographic level over which to intervene to reduce health inequalities. This cannot be achieved by considering only predefined administrative areas as “neighbourhoods” [1, 2]. While this is known to be a strong limitation of prior research of neighbourhood effects on health [3, 4], only few alternatives have been proposed, see [5]. Only limited efforts have been devoted to understanding and explaining the parameters of spatial distributions of health outcomes based on data from geocoded individuals [6–8].

The presence of spatial autocorrelation is an indication of some form of spatial processes affecting the analysed outcome. “Neighbourhood effects” refers to the effects of a range of neighbourhood characteristics, e.g. environmental characteristics like noise or air pollution, economic characteristics like unemployment, or institutional characteristics like access to schools, public transport, and green space, or factors pertaining to the social structure in a neighbourhood. However, spatial autocorrelation can indicate the presence of neighbourhood effects but also of compositional effects (similar people tend to live close to each other). To include the latter we use the umbrella term spatial effects. As a preliminary step before disentangling compositional and neighbourhood effects, methods to assess unmeasured spatial effects are a useful tool to show the existence and magnitude of compositional and neighbourhood effects.

A possible approach to estimate unmeasured spatial effects without having to predefine neighbourhoods is to model the correlation structure of individual health outcomes. This approach has been described in [9] and consists of fitting an exponential model to the so called semi-variogram [10]. This is a way to estimate the spatial correlation structure at an individual level and provide a measure of the proportion of the total variance which is spatially structured as a measure of the strength of the spatial effect (both neighbourhood and compositional effects) and a spatial range which describes how far reaching the spatial structure is, i.e. the distance at which a health outcome of two persons geocoded at their residence is no more correlated. In this way the method provides a measure of the size of the small-area scale over which the health phenomenon of interest varies over the local spatial space, which is an important parameter of the geographic distribution of the outcome. This approach necessitates the geo-coordinates of the place of residence.

Some evidence of a relationship between place of residence and cardiovascular risk factors exists. For example limited possibilities to perform physical activities in a neighbourhood may lead to a more sedentary lifestyle, which in turn may lead to a higher body mass index (BMI) [11, 12]. The social structure could as well play a role in the way a neighbourhood may affect the aforementioned risk factors with some aspect of social cohesion having a protective effect on acute myocardial infarction mortality [13]. Body mass index has been shown to mediate the effects of neighbourhood characteristics such as population density and neighbourhood education on blood pressure [14]. A spatial correlation structure appears to be associated with some cardiovascular risk factors: in prior studies outcomes including obesity, high total cholesterol (TC) and low high density lipoprotein (HDL) showed spatial autocorrelation with a much stronger effect for obesity than for TC or HDL [15]. Therefore we chose to model the correlation structure of cardiovascular risk factors as an example of the possibilities offered by the ego-centred approach to investigate unmeasured local spatial effects on health outcomes in comparison to the multilevel approach based on administrative units.

The aim of this methodological work is to show how the ego-centred spatial correlation structure approach compares to the usual administrative neighbourhood approach using multilevel modelling to show the presence of unmeasured spatial effects. We analyse the presence of unmeasured spatial effects on cardiovascular risk factors in three different European cities of different sizes and population densities, by modelling the spatial correlation structure of individual outcomes as well as fitting a multilevel model to estimate the intra-class correlation using available administrative units.

## Material and methods

### Material

Three datasets were available for analyses:

1) Data from the “Residential Environment and CORonary heart Disease” (RECORD) study, an epidemiologic cohort study conducted in the Paris Ile-de-France region in 2007 and 2008 (first wave) with 7290 participants from 1914 neighbourhoods. Participants between the age of 30 to 79 years were recruited in four health centres, where working and retired employees and family members can get an extensive free preventive medical check-up every five years [16]. Addresses of participants were geocoded. The primary aim of the study was to describe social and spatial disparities in health and analyse the effects of geographic life environments on health. For more details see [16].

2) Data from the “Gesundheit von Babys und Kindern in Bielefeld” (BaBi) study, a birth cohort study conducted in Bielefeld, Germany from 2013 to 2016 (first wave) with 977 participating women 18 to 49 years of age living in one of 84 statistical districts of the city of Bielefeld around the time of birth [17, 18]. Questionnaire data collected by computer assisted personal interviewing were linked to routine data (data related to the mother, the newborn and data on pregnancy and delivery) collected by the hospitals. Only data relating to the mothers were used here.

3) Data from the longitudinal “Dortmund Health Study” (DHS) [19]. The baseline assessment was conducted in 2003/2004, and 2291 participants (response proportion 67%), aged 25 to 74 years and living in one of 62 statistical districts of the city of Dortmund, Germany, either filled out a mailed questionnaire (*N* = 979) on socio-economic status and subjective health, or attended the study centre (*N* = 1312) to answer these questions in face-to-face interviews and receive a medical examination. For more details see [19, 20]

### Measures

Age in years was available of all studies. Educational attainment was measured by the highest graduation level and was classified into the three categories. Different classification systems for education were used in the three studies (school vs. school and occupational education combined; for details see additional Table 1). For all studies the household income could be categorised as < 2000 €, < 4000 €, and > =4000 €.

**Table 1:** Determinants (BMI analyses taken as example) and outcomes

Determinants			
	Record Study	BaBi Study	DHS
**Sex**			
Female	2452 (34.4%)	807 (100.0%)	505 (52.4%)
Male	4685 (65.6%)	–	459 (47.6%)
**Age**			
Mean (SD)	50.2 (11.7)	31.4 (4.8)	52.2 (13.4)
Min. / Max.	30 / 79	18 / 46	26 / 74
**Education**			
Low	548 (7.7%)	113 (14.0%)	465 (48.2%)
Medium	3039 (42.6%)	332 (41.1%)	205 (21.3%)
High	3550 (49.7%)	362 (44.9%)	294 (30.5%)
**Income**			
Low	2239 (31.4%)	182 (22.6%)	420 (43.6%)
Medium	2466 (34.6%)	399 (49.4%)	435 (45.1%)
High	2432 (34.1%)	226 (28.0%)	109 (11.3%)
Outcomes			
**BMI**			
Mean (SD)	25.5 (4.2)	24.5 (5.4)	26.4 (4.6)
Min./Max.	14.3 / 53.7	16.0 / 59.7	13.9 / 49.2
N	7137	807	964
**Systolic Blood Pressure**			
Mean (SD)	128.0 (17.5)	116.1 (12.4)	141.0 (21.3)
Min./Max.	75 / 234	85 / 160	93 / 226
N	7021	393	1232

In the RECORD study, BMI (respectively height and weight) was measured during health examination in the study centres. The DHS study provided self-reported data for height and weight at baseline. In the BaBi study, medical data were extracted from the maternity cards. Pre-pregnancy BMI was estimated based on height and weight measured and documented by medical doctors during the first antenatal care visit which, on average, took place during the first trimester. Women do not gain more than 0.5–2 kg in weight during the first trimester, which corresponds to normal fluctuations in body weight [21].

Systolic blood pressure was measured at the left arm and the maximum pressure was recorded (RECORD study), was measured during first antenatal care visit (BaBi study) or the mean of two measurements was recorded (DHS study for those who attended the study centre).

Participants with missing values in one or more variables were excluded from analyses. Therefore, the total number of participants differs according to the outcome analysed.

For the BaBi Study, the addresses of the participants were geocoded by the authors. After a geo-masking process to protect privacy of participants, the coordinates were temporary linked with the study data. For the other studies, geo-coordinates were provided by the data owners.

### Statistical analysis

The parameters for the spatial correlation structure are obtained using an exponential model for the so-called semi-variogram [10]. The semi-variogram is a way to model the correlation structure between individual (health) outcomes collected from spatially located observations by fitting a parametric exponential model that provides an estimate for a distance H (the practical range) such that two persons separated by a distance greater than H will be, on average, uncorrelated. The total variance of the outcome can be seen as the sum of the so called partial sill and the nugget effect. The nugget effect is the part of the variance which is not spatially structured and the partial sill the one which is spatially structured. We take the relative structure variability (RSV) which is the ratio between the partial sill and the total variance (partial sill + nugget effect) as an indicator of the strength of the spatial correlation structure. For more details of the application of the semi-variogram method to health data, see [9].

The residuals of CVD risk factors were obtained from a linear regression model including age, sex, household income and educational attainment using the SAS procedure” mixed” first not taking the administrative units into account then fitting a random effect model for the effect of administrative units. The parameters of exponential models for the semi-variogram of those residuals were obtained using the SAS procedure “variogram”. Intra-class correlation coefficients were obtained to measure the correlation within administrative units. There are alternative parametric models for semi-variogram which have been used in other contexts [22] and which may provide a more accurate fit for empirical semi-variograms in general [23]. The aim here is not however to propose the best fit for the semi-variogram but an assessment of the presence of unmeasured neighbourhood effects. The exponential model does fit health data reasonably well and provides such measure. The statistical software SAS 9.4 was used for all analyses.

## Results

Participants of the BaBi study are about 20 years younger on average than the participants of the RECORD and the DHS studies and are all pregnant women. According to our classification the educational attainment is highest in Paris (RECORD) and lowest in Dortmund (DHS): the proportionally largest low-income group is found in Dortmund (Germany) and the largest high-income group in Paris (France). The participants of the BaBi study (comparatively young study group) have the lowest mean BMI value (mean 24.5, SD: 5.4), and the difference between participants of the RECORD study and the DHS study is small (mean BMI (SD): 25.5 (4.2) vs. 26.4 (4.6)). A similar picture emerges for mean systolic blood pressure (Table 1).

The spatial distributions of participants were plotted using the BMI data of the three studies. The figures can be found in the Additional file 2. While for all studies some form of data clustering is seen this is mostly visible for RECORD in which only selected areas of the Paris region participated in the study.

**Fig. 1:**
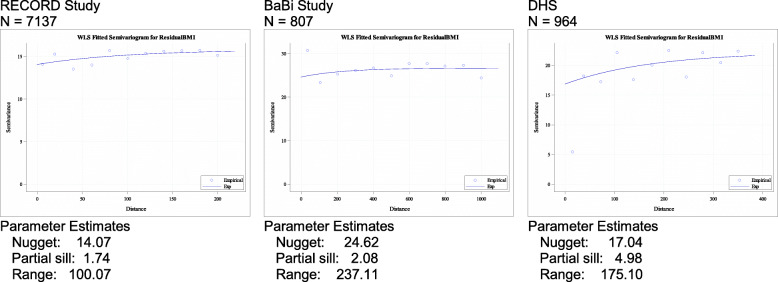
Weighted least squares fitted semi-variograms for residual BMI (adjusted for gender, age, income and educational achievement) of the RECORD, BaBi and DHS studies

Figure 1 shows the semi-variograms fitted with weighted least squares for the residuals of a regression model for BMI for each of the three studies. A correlation structure is seen with ranges from 100 m for RECORD to 237 m for BaBi. While the exponential model (line) fits the RECORD data well, the semi-variogram shows more variability for the BaBi and DHS study due to smaller sample sizes. Adjusting for the administrative units does explain only a small part of the correlation structure of residuals (from 11 to 7% spatially structured in Paris or unchanged in Dortmund) and the ICCs were relatively small (0.05 Paris and 0.04 Dortmund and 0.02 Bielefeld). The difference in ICC can be explained by the difference in administrative units between cities. The results are presented in Table 2.

**Table 2:** Parameters estimates for the semi-variogram of BMI and blood pressure as well as intra-class correlations obtained by fitting multilevel models

	Record Study	BaBi Study	DHS
**BMI**			
ICC	0.0429	0.0237	0.0379
RSV% (without / with administrative units)	11.04 / 7.36	7.77 / 6.69	22.61 / 22.75
**Bloodpressure**			
ICC	0.1605	0.0231	0.0110
RSV% (without / with administrative units)	16.49 / 13.47	3.59 / < 0.01	20.04 / 20.04

**Fig. 2:**
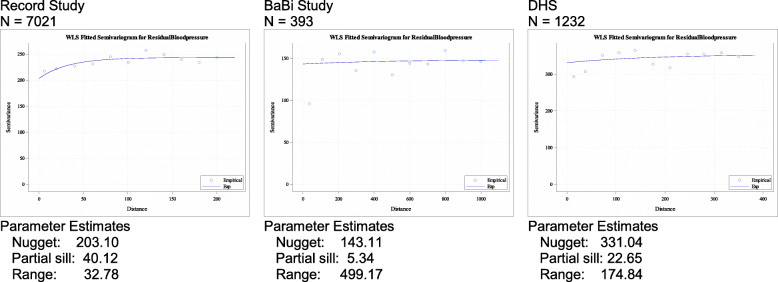
Weighted least squares fitted semi-variograms for residual systolic blood pressure (adjusted for gender, age, income and educational achievement) of the RECORD, BaBi and DHS studies

The results of the semi-variograms for residual systolic blood pressure are similar to the results for BMI but show a weaker structure with smaller RSV. The proportion of residual variability which is spatially structured (RSV) varies from 8 (Bielefeld) to 11% (Paris) and 24% (Dortmund) for BMI and from 4% (Bielefeld) to 6% (Dortmund) and 16% (Paris) for blood pressure (Fig. 2). Adjusting for the administrative units did explain only a small part of the correlation structure of residuals (from 16 to 13% spatially structured in Paris or unchanged in Dortmund) and the ICCs were relatively small (0.03 Paris and 0.01 Dortmund and 0.003 Bielefeld). The differences in ICC can be explained by the differences in administrative units between cities. The results are presented in Table 2.

Table 3 shows a range of data relative to the estimation of the spatial autocorrelation structure. A good estimation of the RSV requires a sufficient number of pairs of observation at small distances. We also present the estimates of population variances as well as residual variances from the data to show how much is explained by the variables in the regression model. Moreover the estimated sill should be ideally equal to the residual variance. In our three examples two sills are slightly underestimating the residual variance and one slightly overestimates it.

**Table 3:** Data relative to the estimation of the correlation neighbourhood

	Record Study	Babi Study	DHS
Population density inhabitants per qm^2^	3763 (Unité urbaine de Paris)	1290	2091
Number of pairs (BMI data)			
0 - 20 m	362	8	7
0 – 50 m	1210	78	56
0 – 100 m	3788	234	195
**Variance**			
BMI	17.51	29.56	21.11
Residual BMI	16.52	27.82	18.89
**Sill (estimated)**	15.81	26.70	22.02
**Variance**			
SBP	304.57	154.96	452.72
Residual SBP	255.69	151.39	343.87
**Sill (estimated)**	243.21	148.44	353.70

## Discussion

Estimating the parameters of the spatial autocorrelation structure for two cardiovascular disease risk factors (BMI and systolic blood pressure) in three European cities (Paris, Dortmund and Bielefeld) has shown that after adjusting for age, sex, educational attainment, and income, unmeasured spatial effects (comprising potential influences of both environmental factors and compositional characteristics) could be found consistently across cities for BMI and to a lesser extend for blood pressure by modelling the individual correlation structure of health outcomes using a semi-variogram approach. The proportion of residual variability which is spatially structured varies from 0.08 to 0.23 for BMI and from 0.04 to 0.20 for blood pressure. We compared this approach to estimating the intra-class correlation obtained by fitting a multilevel model for available administrative units. The individual spatial correlation approach provides much stronger evidence of spatial effects than the multilevel approach even for small administrative units like the ones available for the RECORD study. Only a small part of the correlation of residuals was explained by adjusting for the correlation within administrative units (from 0 to 4 percentage points).

In a subsequent step, analyses would have to include further explanatory factors and examine whether they explain out the spatially structured variability in the outcomes. Multilevel regression based administrative units can complement the individual approach as the administrative unit can actually explain some of the correlation structure seen as in our analysis. But also some explanatory variables may only be available in aggregated form at neighbourhood level.

A limitation of the method used is the need for a sufficient number of neighbours available at small distances for each observation. Therefore, when planning a study, the population density and the spatial distribution of study participants should be carefully examined. While in Paris we had just under 3800 pairs of neighbours at less than 100 m, we had 234 in Bielefeld and 195 in Dortmund (Table 3). Simulations have shown that reliable and precise results can be obtained for Paris while less precision can be expected for Bielefeld and Dortmund [9]. In principle other measures of proximity could be used (e.g. micro data identifier). It would then be necessary to transform these measures in distances (in a mathematical sense) which may be tricky and it may be difficult to deal with observations within the same unit.

While a part of the spatial effects that were identified may be due to compositional effects (similar people tend to live close to each other) this does not diminish the potential relevance of estimating the spatial range of correlation. We have controlled for any structural differences in sex, age, educational attainment, and income, which are common predictors of cardiovascular risk at individual level. While blood pressure and BMI are - besides their age dependency - mostly lifestyle dependent and correlated with each other, BMI is also influenced by social norms which are present in the immediate neighbourhood which may be explaining the stronger spatial correlation. Also obesogenic neighbourhood factors (fast food availability, lack of green spaces) may play a role [24].

A strength of this work is the use of three datasets covering samples from three different European urban areas. This allowed us to estimate consistently the parameters for spatial correlation structure of cardiovascular risk factors across cities. At the same time, this was also a limitation because we could not control for many individual variables due to the difficulty to harmonize between cities. However, we have been able to control for major predictors of cardiovascular risk: age, sex, educational attainment, and income, the latter being known to be strongly spatially correlated.

In conclusion we have shown how modelling the spatial correlation structure at individual level using semi-variograms is a useful tool to establish the existence of unmeasured spatial effects on health outcomes with the example of cardiovascular risks factors providing more precise evidence than the multilevel approach with more potential for application. This method can be used alongside existing methods for the study of spatial or neighbourhood effects on health when geo-coordinates of addresses are available.

## Supplementary Information

**Additional file 1.**

**Additional file 2.**

## Data Availability

This is a secondary data analysis. Therefore the data owners must be contacted regarding data availability. Record Study: Basile Chaix (basile.chaix@iplesp.upmc.fr). BaBi Study: Oliver Razum (oliver.razum@uni-bielefeld.de) and Jacob Spallek (jacob.spallek@b-tu.de). DHS: Klaus Berger (bergerk@uni-muenster.de).
