# Supplementary material for: A method to define the relevant ego-centred spatial scale for the assessment of neighbourhood effects: the example of cardiovascular risk factors
Source: BMC Public Health. 2021 Jul 7;21:1346. doi: 10.1186/s12889-021-11356-w (PMC8265054; doi:10.1186/s12889-021-11356-w)
Supplement: Supplementary file 1 — Additional file 1. [file 12889_2021_11356_MOESM1_ESM.docx]

| **Additional table 1:** Defined categories of education and household income | | | |
| --- | --- | --- | --- |
|  | **Record** | **BaBi** | **DHS** |
| Low education | does not read or write French | No degree | No degree |
|  | no diploma | Student | Elementary school |
|  |  | In vocational training | Secondary school |
|  |  | Other | Other |
|  |  |  |  |
| Medium education | finished up to grade 10 in high school | Technical Collage | Secondary School Graduation |
|  | end of high school (+2 years vocational training) | Master School |  |
|  |  |  |  |
| High education | category above + 2 years tertiary education | Bachelor, Master  or higher degree | High School or higher degree |
|  | Bachelor, Master or higher degree |  |  |
|  |  |  |  |
|  |  |  |  |
| Low household income | <2000 € | <2000 € | <2000 € |
| Medium household income | <4000 € | <4000 € | <4000 € |
| High household income | >=4000 € | >=4000 € | >=4000 € |
|  |  |  |  |
| BMI (height and weight) | measured | measured | self-reported |
|  |  |  |  |
| Systolic blood pressure | measured | measured | measured |
|  |  |  |  |
