# Supplementary material for: A method to define the relevant ego-centred spatial scale for the assessment of neighbourhood effects: the example of cardiovascular risk factors
Source: BMC Public Health. 2021 Jul 7;21:1346. doi: 10.1186/s12889-021-11356-w (PMC8265054; doi:10.1186/s12889-021-11356-w)
Supplement: Supplementary file 2 — Additional file 2. [file 12889_2021_11356_MOESM2_ESM.zip › 11 - Additional figure 1.docx]

lat = latitude, lon = longitude, BMI = Body Mass Index

Additional figure 1: Spatial distribution of BMI observations in the RECORD Study
